# Case Report: Percutaneous closure of patent ductus arteriosus with the KONAR-MF™ device: initial experience and results at 6 months in a tertiary center

**DOI:** 10.3389/fped.2026.1840971

**Published:** 2026-06-01

**Authors:** Alex Ismael Catalán Cabrera, Mónica Karem Medina Durand, Karen del Rosario Condori Alvino, Cesar Augusto Puma Laura, Norma Maribel Portilla Marroquin

**Affiliations:** Department of Pediatric Cardiology, Instituto Nacional de Salud del Niño San Borja, Lima, Peru

**Keywords:** cardiac catheterization, Doppler echocardiography, patent ductus arteriosus, septal occluder device, ventricular septal defect

## Abstract

**Background:**

Patent ductus arteriosus (PDA) results in pulmonary overcirculation and, depending on ductal size and patient age, may lead to symptoms of congestive heart failure and the development of pulmonary hypertension, among other complications. Percutaneous closure is currently considered the first-line option in most patients, depending on clinical and anatomical characteristics. Approved PDA occlusion devices are tailored to specific ductal morphologies; therefore, off-label devices such as muscular ventricular septal defect occluders, vascular plug II, or atrial septal defect occluders have been used in selected patients. With the advent of newer devices, and particularly the KONAR-MF™, a broad range of PDA morphologies can be treated, including in patients weighing <6 kg.

**Objectives:**

To evaluate the clinical and epidemiological characteristics of patients in whom PDA was closed using the off-label KONAR-MF™ device and to report outcomes at 6 months postprocedure.

**Method:**

This was a retrospective, single-center, descriptive observational case-series study with a 6-month follow-up. All patients who used the KONAR-MF™ device between 2024 and 2025 and met the inclusion and exclusion criteria were included in this study.

**Results:**

Seventeen patients were analyzed. The median age was 1 year and the median weight was 11 kg. Female sex accounted for 58.8% of cases. Functional class III was present in 58.8% of patients. Severe pneumonia was the reason for admission in 23.5% of patients. Down syndrome was recorded in 17.6% of patients. According to the Krichenko classification, 47.1% of PDAs were type C, and 11.7% were associated with a ventricular septal defect. The median minimum ductal diameter was 5 mm and the median ductal length was 8 mm. The median pulmonary artery pressure was 25 mmHg, median fluoroscopy time was 9.7 min, and median radiation dose was 46 mGy/cm^2^. Transthoracic echocardiography combined with fluoroscopy was used in 11.8% of patients. The antegrade approach was used in 64.7% of procedures. The most frequently used device sizes were 6/4 and 12/10. On serial echocardiography, a residual shunt was detected in 23.5% of patients, and it had resolved in all of them by 6 months.

**Conclusion:**

Despite the small sample size of this study, the findings suggest that the KONAR-MF™ device, when used off-label, may be a valid alternative in complex anatomies or in underweight patients.

## 1 Introduction

The ductus arteriosus is a fetal vascular structure that connects the aorta to the pulmonary artery and plays a crucial role in fetal circulation. It typically closes spontaneously after birth; failure of closure within the first months of life results in a condition known as “patent ductus arteriosus (PDA).” In Peru, PDA represents the second most common acyanotic congenital heart disease after ventricular septal defect (VSD) in children under 1 year of age (1).

When the ductus remains open, pulmonary hyperflow results, and, depending on the size of the ductus and the age of the patient, symptoms of congestive heart failure (2), risk of pulmonary hypertension (3), and risk of endarteritis (4) occur.

The first percutaneous closure of PDA was performed by Porstmann et al. (5) in 1967, and the first cases in Peru were reported in 1997 (6). Since the introduction of dedicated PDA occlusion devices in 2001, these devices have been used worldwide, with reports of their use in Peru since 2009 (7).

With advances in transcatheter techniques, a variety of occluders have been used for PDA closure (8), including both approved and off-label devices, chosen according to ductal morphology, patient weight, degree of pulmonary hypertension, and other clinical considerations. In our setting, some patients are referred from high-altitude regions and may present with larger PDAs, which can pose additional challenges for transcatheter closure. García-Montes et al. (9) in Mexico reported the use of atrial septal occluders for selected PDAs, whereas Garay et al. (10) in Chile used the Vascular Plug II (11), supporting the safety and effectiveness of transcatheter PDA closure. Salam et al. (12) reported the use of muscular ventricular septal defect occluders in infants and small children with tubular PDAs.

Weight less than 6 kg represents a relative contraindication for percutaneous closure of PDA, due to the possibility of aortic or left branch occlusion and even peripheral vascular injury; however, Gruenwald Gronier et al. (13) show that in this group of patients, the KONAR-MF™ device could be safe and effective in the percutaneous closure of PDA.

The KONAR-MF™ device has been used not only in the closure of PDA but also in other pathologies such as the aortopulmonary window and coronary fistulas, as demonstrated by other researchers (14).

A recent study conducted by Lwin et al. (15) that analyzed a total of 75 patients between 2018 and 2025 in 14 pediatric centers in five countries concludes that the KONAR-MF™ device is effective and safe in the percutaneous closure of PDA.

In our setting, the KONAR-MF™ device is used in the percutaneous closure of VSDs; it began to be used in the closure of PDA because of its characteristics of flexibility, introducer profile, a double-disc design, and retrograde and anterograde release.

## 2 Material and method

### 2.1 Study design and population

This was a retrospective, single-center, descriptive observational study with a 6-month follow-up. The medical records, procedural reports, angiography, and transthoracic echocardiography (TTE) images of the institutional storage system of the 17 patients whose PDA was closed percutaneously with the KONAR-MF™ device between 2024 and 2025 with a follow-up of 6 months after the procedure were reviewed.

### 2.2 Indication for PDA occlusion

Indications for transcatheter PDA closure included poor weight gain, recurrent respiratory infections, and small PDAs without significant left ventricular volume overload, to prevent infective endocarditis. All PDA cases were reviewed in a multidisciplinary cardiology–cardiac surgery conference, and patients selected for percutaneous closure were scheduled for the procedure.

### 2.3 Device selection and change in practice

Ductal morphology was assessed using the Krichenko classification: type A (conical), type B (window), type C (tubular), type D (complex with multiple constrictions), and type E (elongated).

In our setting, only approved PDA occlusion devices are available, and they are primarily used for Krichenko type A PDAs. Given our prior experience using the KONAR-MF™ device for VSD closure and its technical characteristics, this device was adopted as a primary strategy, initially for type C and type E PDAs, in which the use of standard duct occluders was expected to be suboptimal. Subsequently, its use was extended to other ductal types depending on device availability.

The KONAR-MF™ is a low-profile, self-expandable, double-disc device made of a double-layer nitinol wire mesh (144 nitinol wires). It consists of two discs connected by an articulated, expandable, and conical waist. Its flexible waist and soft mesh allow high conformability to the target defect. The device can be deployed via either a retrograde or an antegrade approach. It is available in eight sizes (5/3, 6/4, 7/5, 8/6, 9/7, 10/8, 12/10, and 14/12 mm). In the four largest models, the waist is sewn with a polytetrafluoroethylene (PTFE) membrane using nylon threads. The device is inserted through a delivery sheath, with sheath sizes ranging from 5 to 7 Fr.

The choice between the antegrade and retrograde approaches was individualized according to the patient's weight and the morphology of the ductus arteriosus. The antegrade approach was prioritized in infants weighing less than 10 kg. Conversely, the retrograde approach is generally used in patients weighing more than 10 kg and in patients with small type E ductus arteriosus, where the antegrade approach is more difficult to use, and the retrograde approach provides a more direct route for device placement, reducing fluoroscopy time.

### 2.4 Procedure details

The procedures were performed in a catheterization laboratory, using a Siemens Artis Zee biplane angiograph and a General Electric S 70 echocardiograph. All procedures were performed under general anesthesia.

The angiographic and/or TTE minimum ductal diameter was measured, and at least 2 mm was added to select the diameter of the pulmonary (smaller) end of the device. A delivery sheath was selected according to the chosen device size.

Cefazolin (50 mg/kg/dose) was administered prior to the procedure, and unfractionated heparin (100 IU/kg) was administered after venous/arterial access under ultrasound guidance. Aortography was performed using a 4/5 Fr pigtail catheter in right anterior oblique (RAO) 30°/0° and left anterior oblique (LAO) 90°/0° projections to assess ductal size and morphology. In patients in whom the procedures were performed without contrast and with venous access only, final PDA measurements were obtained by TTE.

After confirming the size and shape of the PDA, a 0.035 in. × 260 cm curved hydrophilic guidewire was placed in the abdominal aorta with a 4/5 Fr right coronary catheter, and if it was only arterial access, the guidewire was positioned as passing from the aorta through the PDA in the pulmonary trunk. After the guidewire was positioned, a long introducer sheath was selected according to the KONAR-MF™ device, leaving it positioned in the thoracic aorta or pulmonary trunk according to the access route. Subsequently, the KONAR-MF™ device was advanced and deployed under angiographic and transthoracic echocardiographic guidance to verify its correct position (Supplementary Video S1).

In all patients, the position of the KONAR-MF™ device, the presence of residual shunts, and whether there was any obstruction of the aorta or left pulmonary branch were corroborated by TTE.

The first four patients had severe pneumonia on mechanical ventilation and returned to the intensive care unit (ICU) to continue their treatment and management, while the rest of the patients were admitted as outpatients scheduled for percutaneous closure of PDA. The first patient had a history of prematurity and was on mechanical ventilation since birth, and although the closure of PDA was successfully performed, the patient died a few weeks later because of chronic infectious and pulmonary complications. The remaining patients were followed up for 6 months postprocedure with serial echocardiographic controls.

### 2.5 Postprocedural monitoring and follow-up regimen

Follow-up was conducted after discharge at 1 week, 1 month, 3 months, and 6 months. However, according to institutional protocol, the patients continue to be followed up annually.

### 2.6 Study variables

The study variables were age, sex, weight, genetic syndrome, history such as prematurity, infectious comorbidities, functional class prior to closure and at 6 months after closure, type of ductus arteriosus according to Krichenko classification (16), associated heart defect, size of the pulmonary end and length of the ductus arteriosus, pulmonary pressure recorded during the procedure, KONAR-MF™ device number used, if fluoroscopy/echocardiography or both were used, the procedure time and radiation dose, peripheral accesses, use of contrast, immediate residual shunt and at 6 months of follow-up, reinterventions, and left pulmonary branch occlusion.

### 2.7 Statistical analysis

Continuous variables were presented as mean ± standard deviation or median with interquartile range (IQR), as appropriate. Categorical variables were expressed as frequencies and percentages. The percentages were rounded to 1 decimal place.

### 2.8 Ethical aspects

This retrospective study met all the requirements contained in the WHO code of ethics (Declaration of Helsinki) and was reviewed and approved by the ethics committee of the National Institute of Children's Health, San Borja, and all patients had copies of the informed consent form of the procedure signed by their parents. Written informed consent was obtained from the parents or legal guardians for the publication of any potentially identifiable images or data included in this article.

## 3 Results

**Figure 1:**
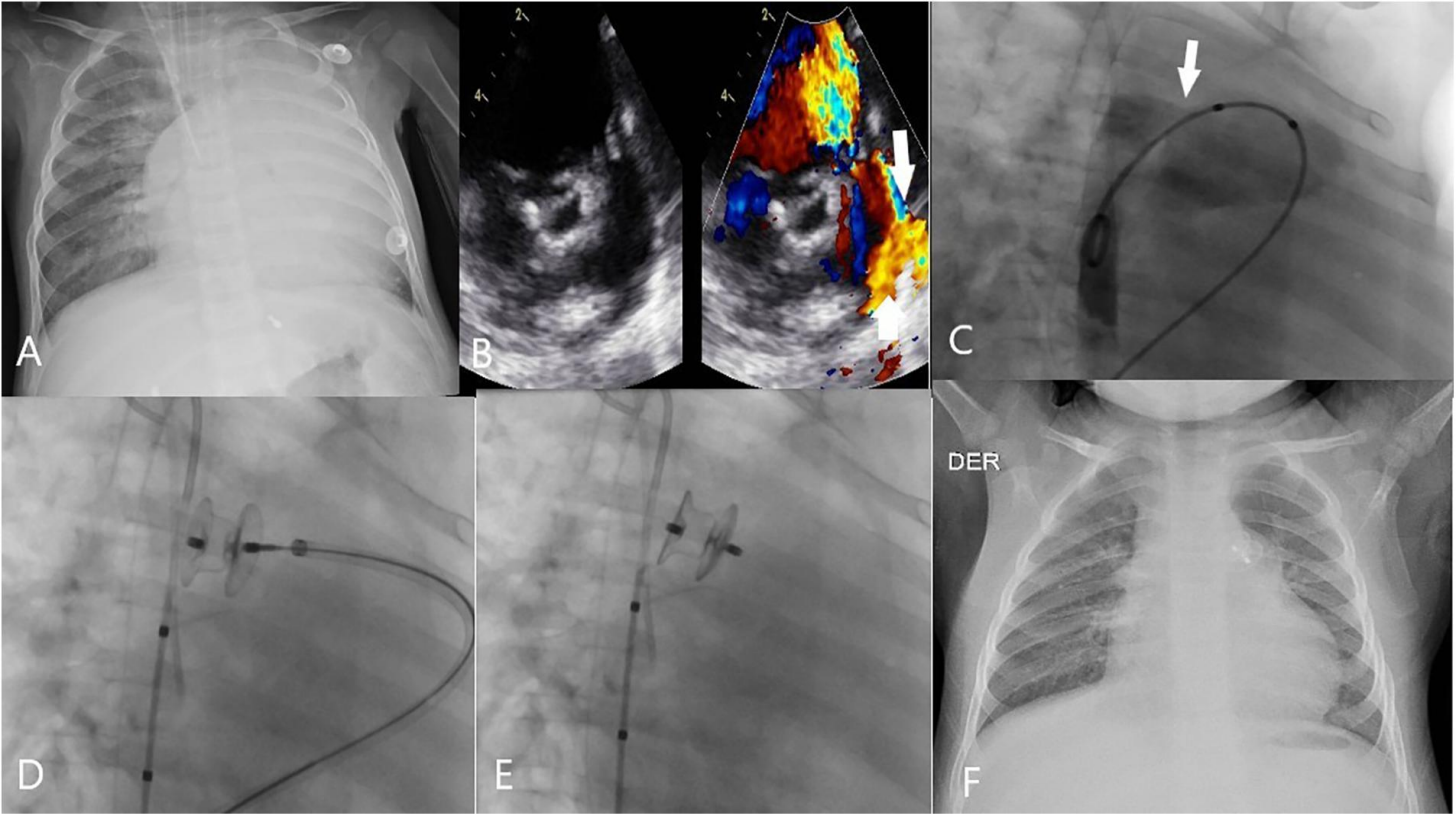
Percutaneous closure of patent anterograde ductus arteriosus with a KONAR-MF™ 12/10. **(A)** A chest X-ray prior to percutaneous closure of PDA. **(B)** TTE where we visualize the PDA (white arrow). **(C)** Aortography in LAO 90°/0° projection showing tubular patent ductus arteriosus (white arrow). **(D)** Deploying the device at the aortic level. **(E)** The device released. **(F)** A chest X-ray 6 months after percutaneous closure of the ductus arteriosus.

**Figure 2:**
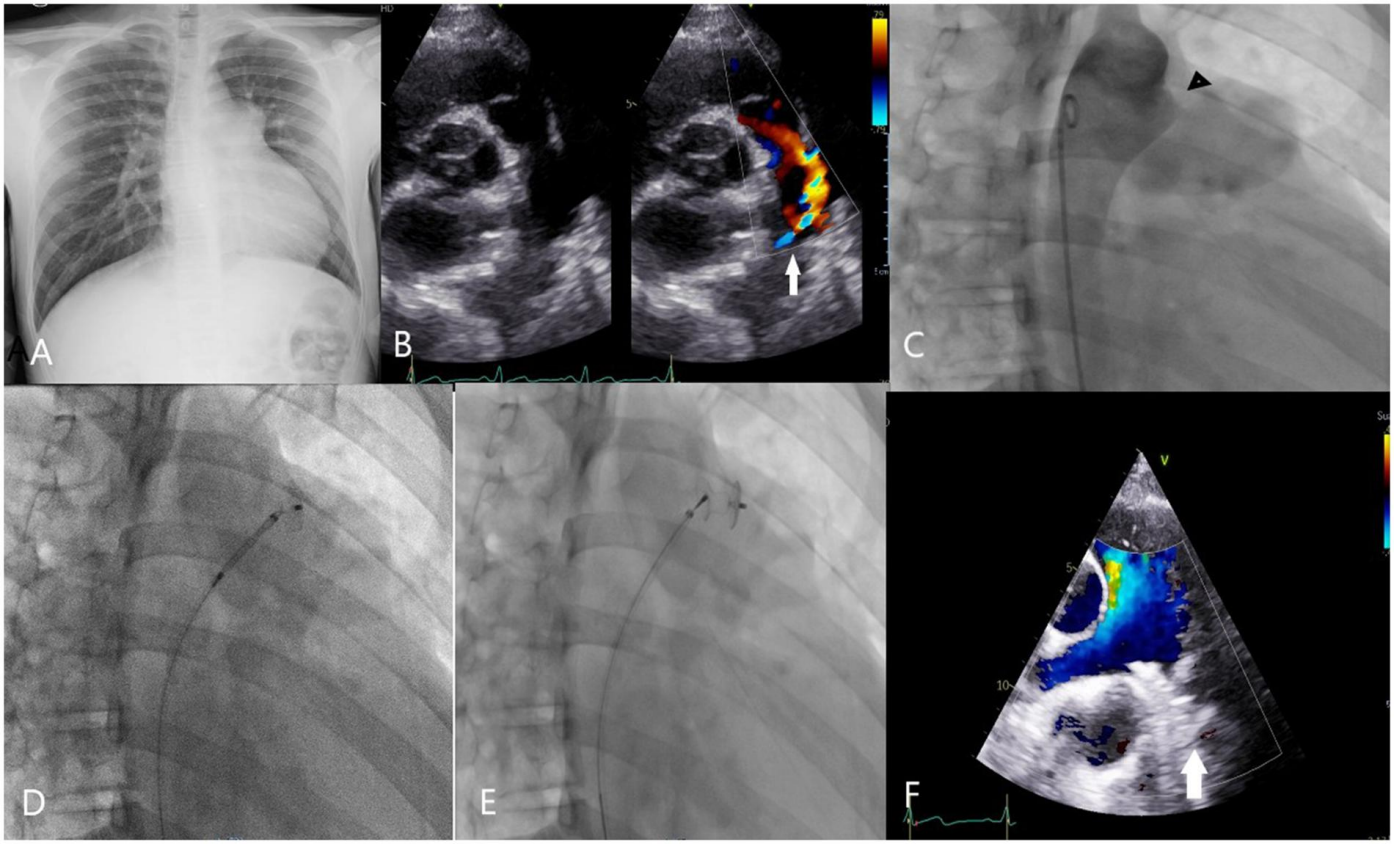
Percutaneous closure of retrograde patent ductus arteriosus with a KONAR-MF™ 6/4. **(A)** A chest X-ray prior to percutaneous closure of PDA. **(B)** TTE where we visualize the PDA (white arrow). **(C)** Aortography in LAO 90°/0° projection showing PDA type E (black arrow). **(D)** Deploying the first disc of the KONAR-MF™ device. **(E)** Deploying the second disc of the KONAR-MF™ device prior to delivery. **(F)** Echocardiography after immediate percutaneous closure of PDA.

A total of 17 patients in whom percutaneous closure of PDA was performed with a KONAR-MF™ device were analyzed. The median age was 1 year, the median weight was 11 kg, and 58.8% of the patients were female. According to the Krichenko classification, type C was found in 47.1% of patients, type E in 29.4%, type A in 17.6%, and type B in 5.9%. With regard to functional class, 58.8% of patients presented with functional class III and 41.2% with functional class I. A total of 11.7% of patients had an associated ventricular septal defect, which was also closed in the same procedure with the KONAR-MF™ device. A total of 23.5% of patients presented with decompensation and severe pneumonia; 17.6% had Down syndrome. Only one patient, representing 5.9%, had a history of prematurity, prolonged hospitalization, prolonged intubation, and died from respiratory and infectious complications unrelated to the procedure (Table 1, Figures 1, 2).

**Table 1:** General characteristics.

Clinical and demographic variables	Patients *n* (17)
Age (years), median (IQR)	1 (0.9–7)
Weight (kg), median (IQR)	11 (5–25)
Female sex	10 (58.8%)
PDA type	
C	8 (47.1%)
E	5 (29.4%)
A	3 (17.6%)
B	1 (5.9%)
Functional class	
I	7 (41.2%)
III	10 (58.8%)
Associated defects (VSD)	2 (11.7%)
Comorbidities (severe pneumonia)	4 (23.5%)
Genetic syndrome (Down)	3 (17.6%)
Antecedents (prematurity)	1 (5.9%)

IQR, interquartile range; VSD, ventricular septal defect.

The narrowest part of the PDA was 5 mm with a median length of 8 mm. A median pulmonary pressure of 25 mmHg was recorded. The median fluoroscopy time was 9.7 min, with a median radiation dose of 46 mGy/cm^2^. The procedure was performed with fluoroscopic guidance and use of contrast in 88.2% of patients, arterial and venous femoral vascular access was gained in 52.9% of patients, arterial access only in 35.3%, and venous access only in 11.8%. The KONAR-MF™ 6/4 device was used in 29.4% of patients, and 10/12 and 8/10 devices were used in 23.5% of patients. Immediate residual shunt was present in 23.5% of patients. In two patients (11.7%) who also had VSD, percutaneous closure was performed with KONAR-MF™ (Table 2, Supplementary Video S1).

**Table 2:** Characteristics of the procedure.

Procedure variables	Patients *n* (17)
Narrowest part PDA (mm), median (IQR)	5 (3.5–6.0)
PDA length (mm), median (IQR)	8 (5 −9)
mPAP (mmHg), median (IQR)	25 (17–31)
Fluoroscopy time (min), median (IQR)	9.7 (7.2–19.5)
Radiation dose (mGy/cm^2^), median (IQR)	46 (18.2–72.9)
Additional procedure	
VSD closure	2 (11.7%)
Immediate residual shunt	4 (23.5%)
Use of contrast	15 (88.2%)
Guiding method	
Fluoroscopy	15 (88.2%)
Fluoroscopy + TTE	2 (11.8%)
Femoral vascular access	
Venous/arterial	9 (52.9%)
Arterial	6 (35.3%)
Venous	2 (11.8%)
KONAR-MF™ device size	
6/4	5 (29.4%)
12/10	4 (23.5%)
10/8	4 (23.5%)
8/6	2 (11.8%)
7/5	1 (5.9%)
9/7	1 (5.9%)

IQR, interquartile range; TTE, transthoracic echocardiography; mPAP, mean pulmonary artery pressure.

No vascular access complications occurred in the patients. After device deployment, no aortic obstruction or left pulmonary artery branch obstruction was observed. An immediate trivial transdevice residual shunt (17) was observed in four patients (23.5%), including two devices with a PTFE membrane, corresponding to Krichenko type B and type C ductal morphologies, with no evidence of hemolysis on follow-up. No reinterventions were required, and at 6 months, the 16 patients who completed follow-up showed no residual shunt and were grouped in functional class I (Supplementary Video S2).

## 4 Discussion

While it is true that devices have been created specifically for the percutaneous closure of the ductus arteriosus, such as the Amplatzer ductal occluder family with different generations since the late 1990s. Throughout the last two decades, various off-label devices have also been used for the closure of PDA, considering the morphology, patient weight, and degree of pulmonary hypertension, among other things, as these are considered safe and effective devices (17).

Median age and weight were similar to those reported by Lwin et al. in their international series, which included 72 children from 14 centers in five countries (15). In our center, the KONAR-MF™ device was initially used in the first four patients, all of whom were clinically unstable due to severe respiratory infection, had Krichenko type B or type C PDAs, and weighed <6 kg. In these patients, the approved PDA occlusion devices available in our institution are primarily intended for Krichenko type A ducts.

In our series, Krichenko type C PDAs predominated, in contrast to the series by Lwin et al. (15) and Banpurkar et al. (11), in which type A was most frequent. This supports the concept that the KONAR-MF device can be used across a broad spectrum of ductal morphologies. The minimum ductal diameter and ductal length were larger in our cohort than those reported by Lwin et al. (15). The fluoroscopy time was similar to that in other studies. The radiation dose was higher in patients with an associated ventricular septal defect that was treated during the same procedure.

Unlike the series by Lwin et al. (15), which reported a 14% residual shunt, and Banpurkar et al. (11), which reported no residual shunt, in our series, 23.5% of patients (Krichenko type B and type C) had an immediate residual shunt. However, on serial echocardiography, this decreased over time and had resolved in all cases by 6 months. One possible explanation is that the KONAR-MF™ device has a finer nitinol mesh, which may influence the time required for complete thrombosis after deployment.

We consider that the use of echocardiography during the deployment of the device was significant in two patients, as also demonstrated by our group in a previous study (18), avoiding the use of contrast and femoral arterial access.

In our study, the proportion of procedures performed through the antegrade approach was similar to that reported by Lwin et al. (15), whereas the retrograde approach was used more frequently. Considering ductal morphology and patient weight, the retrograde approach may be a valid option in selected cases, potentially reducing procedure time and radiation exposure, as demonstrated by Zhou et al. (19).

No immediate complications, nor any complications during the 6-month follow-up, such as vascular injury, device migration, left pulmonary artery occlusion, or aortic obstruction, were observed. In contrast to the 96.1% success rate reported by Lwin et al (15), our group achieved a 100% success rate, possibly influenced by prior experience, having used this device for VSD closure. Given the flexibility of the KONAR device compared with other devices, this could represent an advantage and reduce the risk of left pulmonary artery or aortic occlusion, as demonstrated in our study.

We suggest that due to its versatility, the KONAR-MF™ device adapts to all morphologies of PDA and can be used even in patients with a weight of less than 6 kg, as demonstrated by other researchers (13, 15, 20, 21).

These findings suggest that the KONAR-MF™ device could be a valid alternative in complex anatomies or in underweight patients, where approved devices for PDA occlusion might not be useful.

### 4.1 Limitations

This study has several limitations such as a small sample size, its retrospective, single-center observational design without a control group, and the fact that the results reflect the experience of a single tertiary center that used the device mainly in complex PDA morphologies such as Krichenko types C and E.

## 5 Conclusion

Percutaneous PDA closure using the KONAR-MF™ device appears feasible in complex morphologies, particularly when approved PDA occlusion devices are not suitable or not available for the specific ductal anatomy.

## Data Availability

The raw data supporting the conclusions of this article will be made available by the authors without undue reservation.

## Supplementary material

Supplementary Video S1
https://vimeo.com/manage/videos/1173630415

Supplementary Video S2
https://vimeo.com/manage/videos/1187844876
